# How Does Digital Leadership Foster Employee Innovative Behavior: A Cognitive–Affective Processing System Perspective

**DOI:** 10.3390/bs14050362

**Published:** 2024-04-25

**Authors:** Pengbin Gao, Yinghui Gao

**Affiliations:** School of Economics and Management, Harbin Institute of Technology, Weihai 264209, China

**Keywords:** digital leadership, psychological empowerment, affective commitment, proactive personality, employee innovative behavior, cognitive–affective processing framework

## Abstract

Employee innovative behavior is crucial for organizations to engage in innovative activities and gain competitive advantages in the context of digital transformation. Despite many studies having focused on the relationship between leadership and employee innovative behavior, the role of digital leadership and the underlying mechanisms for employee innovative behavior remain unclear. Using the cognitive–affective processing system framework, the study investigated the dual mediating role of psychological empowerment and affective commitment between digital leadership and employee innovative behavior and the moderating role of a proactive personality in such relationships. Employing data from 359 employees, the study conducted structure equation modeling to examine the hypotheses. The results show that digital leadership influences employee innovative behavior through psychological empowerment but not affective commitment. Furthermore, a proactive personality does not moderate the direct effect of digital leadership on psychological empowerment and affective commitment or the indirect effect of digital leadership on employee innovative behavior. Theoretical and practical implications are discussed.

## 1. Introduction

Innovation has been seen as a key way for firms to cope with complex and ever-changing environments and to ensure sustainable competitive advantage [1]. In the current context of digital transformation, innovation is more important for firms than ever before [2]. Consequently, as the cornerstone of organizational innovation, employee innovative behavior plays an increasingly important role in helping firms cope with the increased pressure for innovation brought about by digitalization [3]. Thus, how to stimulate employee innovative behavior has become more and more important and urgent in the digital era [4]. Among a wide range of external factors, leadership has always been considered a key antecedent influencing employee innovative behavior. While prior studies have highlighted the importance of different leadership styles in stimulating employee innovative behavior [5,6,7,8,9,10], they have largely ignored exploring the role of digital leadership, as an emerging leadership style, in promoting employee innovative behavior.

Digital transformation has changed the nature and performance of leadership, requiring leaders to apply a new leadership style—that is, digital leadership leads firms to obtain competitive advantages. Digital leadership is viewed as a series of abilities, behaviors, and practices that inspire and motivate employees in the context of digital transformation [11]. Although companies have recognized the importance of digital leadership, the enthusiasm of researchers for this important phenomenon has just been ignited. Overall, studies on digital leadership outline its origins, concepts, characteristics, and other relevant topics related to digitalization.

Research has shown that leadership and its interaction with employees are crucial for the process of change and innovation, but especially for digital transformation [12]. However, research on the impact of digital leadership on employee innovative behavior is still scarce, irrespective of an increasing interest in both concepts. This paper attempts to address several research gaps in digital leadership research. Firstly, digital transformation is an emerging research field, and there is little research discussing the role of digital leadership in the digital transformation processes of firms [13]. Although some studies have been related to digital leadership and employee outcomes, such as job motivation [14] and employee performance [15], discussion on the consequences of digital leadership at the employee level is inadequate. Our study investigates the effect of digital leadership on employee innovative behavior, which enriches the research on the theory of digital leadership. Secondly, although some research has confirmed that digital leadership contributes to employees’ outcomes, the mechanisms and pathways have not been explored. To date, studies have mostly focused on the relationship between digital transformation-oriented leadership behavior and innovative job performance [16], the effect of E-leadership on employee innovative behavior [17], or the direct effect of digital leadership on employee innovative behavior [18,19]. Nevertheless, the existing literature still does not provide a complete and clear answer to the question of how digital leadership influences employee innovative behavior. Thirdly, previous research has found that innovative behavior relies on many individual, group, and organizational factors that are interrelated [20,21]. There are very few studies analyzing the effect of digital leadership on employee innovative behavior, and even less is known about the moderating effects of other factors. Hence, it is very necessary to analyze which factors could moderate the relationship between digital leadership and employee innovative behavior.

In response to these calls for further research, this study adopts the cognitive–affective processing system (CAPS) framework to explore the mediating mechanism between digital leadership and employee innovative behavior. According to the CAPS framework, individual behavior originates from the interaction between situational and cognitive–emotional factors [22]. Specifically, an external situation stimulates cognitive and affective reactions, which, in turn, activate some corresponding behaviors. This study explores psychological empowerment and affective commitment as cognitive and affective mechanisms because previous studies have theoretically justified and empirically tested the mediating role of these two constructs in the relationship between leadership style and individual outcomes [23,24,25,26]. Thus, we believe that the two constructs can be applied to explore how digital leadership promotes employee innovative behavior from cognitive and affective perspectives.

In addition, this study also investigates the boundary conditions that may moderate the relationship between digital leadership and psychological empowerment and affective commitment. According to the cognitive–affective processing system (CAPS) framework, individual traits can explain the relationship between external situations and their cognitive as well as emotional responses [27]. Previous research has identified a proactive personality as one of the most important personality traits that can interact with leadership style to influence individual outcomes [28,29]. Therefore, this study examines whether a proactive personality moderates the relationship between digital leadership and psychological empowerment and affective commitment.

The rest of the paper is organized as follows. Section 2 reviews the literature and sets up a theoretical framework from which to draw the hypotheses. Section 3 describes the data and research methods to be used to test the hypotheses. Section 4 analyzes the empirical results. Section 5 discusses the results, implications, and limitations, and possible future research directions are revealed. The conclusion is shown in Section 6.

## 2. Theory and Hypothesis

### 2.1. Digital Leadership

Based on leadership and digital transformation, digital leadership is defined as a new paradigm with a different leadership style that exhibits great agility [30]. Digital leadership is defined as the skills, competencies, and leadership styles of the leader to achieve a digitally enabled business model [31]. The combination of digital competence and leadership skills constitutes the characteristics of digital leaders [32]. Digital leadership means thinking differently about business models and strategies and is a process through which firms can realize strategic digitalization success [33]. In a turbulent business environment, digital leaders must articulate a vision and create the conditions to achieve digital maturity [34].

The leadership theory focuses on some typical leadership styles, such as transformational leadership, transactional leadership, and authentic leadership. Transformational leadership transforms followers, prompting them to consider organizational rather than personal interests, boosting their morale, encouraging them to align their personal values with organizational values, calling on them to have an ideal understanding of the organization, and encouraging them to do their best. In contrast, transactional leadership involves exchanges, expectations, and rewards and achieves organizational goals by motivating desired behavior or preventing negative behavior [35]. Authentic leadership promotes the positive psychological capacities of followers and cultivates a sense of unity through transformation and interaction [36]. Previous studies have posited that there are differences and connections between these traditional leadership styles and digital leadership. Digital leadership is viewed as a combination of the transformational leadership style and digital technology [37]. Similarly, digital leadership is considered a combination of transformational leadership, digital knowledge, and digital experiences [38]. Furthermore, digital leadership is considered to have a multidimensional structure, including elements of transformational leadership, transactional leadership, and authentic leadership. The development of digital leadership based on these three types of leadership styles also indicates that, in addition to these characteristics, it is also necessary to cultivate digital thinking and skills to help employees adapt to and adopt digital change [39]. Therefore, attention should be paid to the differences between digital leadership and other leadership styles. Digital leadership should involve a clear digital strategy that combines leadership and culture and always be ready to lead change [40].

In summary, digital leadership can play an important role in driving business change and developing talent complexity in the context of digital transformation [41]. According to the research questions and purposes involved in this study, digital leadership is defined as the combination of leadership style and digital capabilities aimed at optimizing the benefits of digital technologies and developing a digital mindset to inspire and help employees and organizations deal with digital changes and achieve digital business goals.

Furthermore, digital leadership is often linked to other related constructs, such as virtual leadership and e-leadership. Given the diversity of virtual working environments, it is not surprising that leadership in different virtual settings is also discussed under different conceptualizations. Virtual leadership emphasizes managing employees who are geographically dispersed and primarily rely on electronic media for communication and collaboration [42]. Thus, the concept of virtual leadership is considered broader. The concept of e-leadership has been widely discussed since the early 2000s, while the concept of digital leadership is a relatively recent one [43]. E-leadership is usually conceptualized as a social influence process that employs information technology to change employees’ attitudes, feelings, thinking, and behavior, and digital leaders combine leadership skills and digital technologies to assist in decision-making processes [44].

Due to its contribution to developing a wide range of skills and competencies, digital leadership has become a survival factor for businesses in the digital era [45]. Previous studies have investigated the value of digital leadership at different organizational levels, including increasing employees’ performance [46], promoting entrepreneurial team success [47], and elevating firms’ performance [48]. Although many researchers have investigated the issues and impacts of digital leadership from different perspectives [49,50], digital leadership is still under-researched [51].

### 2.2. Employee Innovative Behavior

Employee innovative behavior has been highly regarded for decades. Generally, employee innovative behavior encompasses all individual actions aimed at generating, modifying, communicating, and implementing novel ideas [52]. Therefore, many researchers have defined employee innovative behavior as a multistage and multidimensional concept [53].

Although employee innovative behavior is considered a unique construct, it is often confused with employee creative behavior. This confusion has led researchers to clarify the difference between these two definitions [54]. Primarily, it has long been believed that creativity and innovation are perceived to go hand in hand and are interlinked [55]. However, creativity has traditionally been characterized as the discovery and creation of novel and useful ideas, as opposed to innovation, which has traditionally been defined as the creation and successful implementation of such ideas [56]. Thus, employee creative behavior is a stepping stone to employee innovative behavior [57].

The increasing popularity of employee innovative behavior is not surprising because it has been regarded as an important predictor of outcomes at multiple levels in organizations. Previous studies have indicated that employee innovative behavior is a key driver of employees’ job performance and promotability [58], project team success [59,60], and a firm’s innovation capability and performance [61,62]. Given the increasing importance of employee innovative behavior in gaining competitive advantage and promoting business success, previous research has identified several antecedents relating to attributes of an individual and characteristics of the work environment, including job experience [63], self-efficacy and self-identity [64], engagement [65], identity conflict [66], high-performance work practices [67], and organization justice [68].

### 2.3. Digital Leadership and Employee Innovative Behavior

Previous studies have confirmed that digital leadership plays a crucial role in promoting firms’ innovation. Digital leadership can increase innovative performance [69], strengthen service innovation capacity [70], achieve open innovation [71], elicit responsible innovation [72], and promote radical green innovation [73]. Moreover, digital leadership can positively moderate the effects of digital technology usage and innovation capability [74]. Hence, we believe that there may also be a positive relationship between digital leadership and employee innovative behavior.

Some studies have suggested that digital leaders must adopt multiple roles that play a crucial role in facilitating employee innovative behavior in digital transformation. Based on the competing values framework, the digital transformation leadership framework has identified seven digital leader roles [75]. These different roles require leaders to fulfill corresponding behaviors, including seeking out new opportunities, investigating changes, exploring creative approaches, empowering employees to experiment, sharing relevant information, and so on. Similarly, another study explored five roles of digital leaders and pointed out that digital leaders must make employees open their minds, generate new ideas, and work jointly with others [39]. Undertaking a comprehensive review of the relevant literature, a recent study also identified eight digital transformation leaders’ roles that require leaders to support innovative services and create innovative digital solutions to improve employee experience in the digital workplace [76].

Meanwhile, some research has captured the innovative characteristics of digital leaders. Digital leadership is about innovative behavior [77] and is also an approach that emphasizes innovation, facilitates the introduction of changes and the use of new methods [78], encourages collaboration with employees, and is open to innovation [79]. The innovative characteristics possessed by digital leaders can further contribute to employee innovation. Digital leadership can encourage employees to experiment with new technologies and consider new approaches to solving problems [80]. Digital leadership must provide resources and make structural changes [81] and should guide employees towards a new way of working [82,83]. Digital leadership can create a successful digital workplace to promote employee innovation [84].

Furthermore, recent empirical evidence has demonstrated that digital leadership is critical in increasing employees’ creativity [85,86]. Based on these arguments, we hypothesized the following:

**Hypothesis** **1** **(H1).** *Digital leadership positively affects employee innovative behavior.*

### 2.4. The Dual Mediating Role of Psychological Empowerment and Affective Commitment

#### 2.4.1. Cognitive Path: The Mediating Role of Psychological Empowerment

Psychological empowerment can be defined as a set of motivational cognitions that include meaning, competence, self-determination, and impact, which reflect employees’ orientation toward their job role [87]. Meaning represents the value of individual goal achievement according to predetermined standards. Competence refers to the concept of self-efficacy. Self-determination comprises autonomy, or having the power to initiate and regulate work behavior. Impact means the degree to which an individual believes they can influence others’ work and outcomes [88]. Recent research also has examined the mediating role of employee empowerment in the relationship between digital leadership and organizational performance [89].

By enacting the four cognitions, digital leadership can develop employees’ psychological empowerment. First, digital leadership can build skills to be executed well in a fast-paced and complex environment while creating a growth mindset. Digital leadership is important in motivating employees to be curious, think alternatively, and expand employees’ knowledge through lifelong learning [90]. Similarly, digital leadership can encourage employees’ engagement in change initiatives and can further influence employees’ preferences for learning and mastery [91]. By creating work structures that promote the engagement of employees in digital work, digital leadership could provide dignity through work for those who were previously in the shadows and unable to live a meaningful life [92]. Second, digital leaders can act as role models and pioneers to encourage employees to have a positive attitude toward digital transformation [93]. Digital leaders need to ensure the provision of sufficient and qualified employees [94] and reconcile employees with different degrees of technological aptitude and knowledge [95]. Digital leadership can also instill confidence in employees in risky digital transformation endeavors [96]. Furthermore, digital leadership can help employees learn skills to achieve goals and develop digital capabilities [97]. Third, given the dynamic working structures and employee empowerment, digital leaders retain less power and make decisions in more transparent ways [98]. Digital leadership can help employees achieve goals effectively, actively participate, and successfully overcome the limitations of their working conditions [99]. The transparency and voicing opportunities that digital leadership affords can positively increase employees’ sense of autonomy and allow employees to change their ways of working and monitor their work results [100]. Finally, digital leadership can help cultivate the self-leadership skills of employees and inspire them to reduce resistance to change [101]. Similarly, digital leadership can help dissuade employees from resisting the utilization of different digital technologies and encourage employees to use digital technologies to deal with uncertainty [102]. Digital leadership can help employees stay viable and remove obstacles that may arise during digital transformation [103].

Numerous previous studies have examined the influence of psychological empowerment on employee innovative behavior. Once employees realize that their work is meaningful, they will be committed to it and enthusiastic about it and may also think of innovative ways to use existing resources to solve problems [104]. If employees receive support and repeatedly receive positive assurance about their active commitment to solving complex problems and managing uncertainty, they can persist and focus on achieving innovation goals [105]. Innovative behavior includes trial and error and acceptance of failure as the foundation of learning. The feeling of autonomy provides employees with a chance to repeatedly try new ideas without worrying about being punished or judged for doing so [106]. When employees believe that fulfilling their responsibilities can better serve public interest and improve their well-being, they will use existing resources to think of different innovative ways to complete their work [107].

The CAPS framework explains that employees evaluate external factors that cause them to behave in a certain way. Thus, digital leadership helps distinguish employees of one organization from other organizations and therefore strengthens employees’ self-concept and leads employees to innovative behavior of which they can be proud [108]. Based on the above explanation, we argue that psychological empowerment provides the mechanism through which digital leadership influences employee innovative behavior. Thus, the following hypotheses are proposed:

**Hypothesis** **2** **(H2).** *Digital leadership positively affects psychological empowerment.*

**Hypothesis** **3** **(H3).** *Psychological empowerment mediates the relationship between digital leadership and employee innovative behavior.*

#### 2.4.2. Affective Path: The Mediating Role of Affective Commitment

As one of three dimensions of organizational commitment, affective commitment can be defined as an affective attachment to an organization characterized by shared values, a desire to remain in the organization, and a willingness to exert effort on its behalf [109]. Affective commitment generally requires one to be integrated into interpersonal relationships to maintain and develop them [110]. Recent research has also examined the mediating role of teachers’ commitment in the relationship between principals’ digital transformational leadership and schools’ effectiveness [111].

According to affective event theory (AET), leaders can influence employees’ affect through daily behaviors and emotional expression, thus changing employees’ behavior [112]. There are three reasons why digital leadership can influence employees’ affective commitment. First, digital leadership can create safe, positive, and fair atmospheres for communication and organizational environments that can build on the affective commitment of employees [113] and cultivate a strong sense of collaboration and unity among employees [114]. Digital leadership can create and maintain organizational identity by sharing organizational goals, building a trusting working environment [115], and elevating employees’ commitment [116]. Digital leadership can focus on interpersonal relationships and emphasize trust in employees rather than giving unilateral instructions from the perspective of senior management [117]. Second, digital leadership can consider the employees’ well-being and sustainable development. Digital leadership can build a positive atmosphere for employees to avoid them experiencing isolation, loneliness, and a weakened sense of mission [118]. Digital leadership can create self-sustaining spirals between employees’ productivity and well-being, consider the positive effect of goals and processes on an organization to promote sustainable growth, and inspire employees to engage and overcome barriers [119]. Recent research has examined how digital leadership can help manage employees’ emotional challenges and reduce the negative impact of digital transformation on employees’ health, including isolation, misunderstandings, ambiguity, reduced interpersonal contact, heavy workload, and reduced rest time [120]. Digital leadership also has a visionary and successful social intelligence in directing the emotions of employees [121]. Third, digital leadership can increase employees’ willingness to initiate change. Digital leadership can help resolve differences in opinions, bring employees together, and encourage employees to reach a consensus by negotiating with each other [122]. Digital leadership encourages employees to commit to digital initiatives and understand the nature of their contributions in the long run [123]. Digital leadership creates and communicates organizational vision through continuous motivation and feedback, encouraging employees to actively invest more time and energy into their work [124].

Numerous previous studies have examined the influence of affective commitment on employee innovative behavior. Employees who have an emotional involvement in the organization are willing to improve organizational outcomes by demonstrating innovative behavior [125]. Similarly, it has been suggested that affectively committed employees have a strong identification with organizational values and objectives [126] and are more likely to create and implement novel ideas [127]. In addition, employees with high affective commitment are more likely to exhibit innovative behavior because they are more willing to share ideas and increase social interaction [128]. Furthermore, to thrive, organizations need innovative behaviors from employees to cope with difficulties and gradual changes during turbulent times. Accordingly, highly affectively committed employees are more likely to take risks and thrive on challenges, thereby assisting their organization [129].

The CAPS framework explains that employees experience external factors that cause them to behave in a certain way. Therefore, digital leaders who develop and articulate a common and long-term vision will enhance employee innovative behavior, as employees will have a high degree of motivation to put in extra effort to achieve their vision and develop new methods of problem-solving [130]. Based on the above explanation, we argue that affective commitment provides the mechanism through which digital leadership influences employee innovative behavior. Thus, the following hypotheses are proposed:

**Hypothesis** **4** **(H4).** *Digital leadership positively affects affective commitment.*

**Hypothesis** **5** **(H5).** *Affective commitment mediates the relationship between digital leadership and employee innovative behavior.*

### 2.5. The Moderating Role of a Proactive Personality

A proactive personality is defined as a behavioral tendency to take initiative in various situations [131]. Although people usually respond and adapt to constantly changing situations in the environment, proactive individuals can effectively take initiative and achieve positive results for themselves and their organization [132]. A proactive personality has been expanded to three core components: being future-focused, having self-initiative, and bringing about meaningful changes [133]. Previous empirical studies have shown that a proactive personality is a complex and multidimensional concept which has created many significant and positive outcomes for organizations and individuals [134]. At the same time, previous research has also examined the moderating role of a proactive personality in the relationship between leadership and individual outcomes [135]. Previous research has considered three types of employee proactivity, namely positive framing, sense-making, and relationship building [136]. Therefore, we mainly analyze the moderating effect of these three aspects.

#### 2.5.1. The Moderating Role of a Proactive Personality in the Relationship between Digital Leadership, Psychological Empowerment, and Employee Innovative Behavior

Whereas digital leadership can facilitate psychological empowerment through the four mechanisms discussed, we believe its influence also depends on the characteristics of its followers. First, in terms of positive-framing-related aspects, proactive employees generally show a higher willingness to implement change [137]. Thus, they can see digitalization as an opportunity with great potential to create value [138]. Moreover, positive framing can help employees restructure their knowledge and work and further broaden their cognitions and actions [139]. Second, in terms of sense-making-related aspects, four cognitions in the empowerment framework have a high degree of contextual relevance and should be operated according to actual application scenarios, and thus sense-making can be used to facilitate psychological empowerment [140]. Sense-making is generally based on extracted cues, suggesting that individuals only care about their surroundings [141]. As part of the employees’ environment, digital leadership can build supportive contexts that allow employees to choose adaptive environmental cues when facing complex challenges [142]. Third, proactive employees can actively establish positive relationships with others, thereby enhancing their positive cognitions. Due to establishing good and stable relationships with employees, leaders are more likely to encourage employees to control the consequences of their behavior, clearly communicate organizational goals to employees, promote employee participation in decision-making processes, and share more useful information with employees, thus increasing their levels of involvement and motivation [143,144]. Employees who have a good relationship with leaders can direct their energy to their jobs as a way of carrying out reciprocal behaviors in relationship building [145]. Thus, proactive employees are more likely to understand the work support and participative decision-making provided by digital leaders.

Furthermore, from the leaders’ perspective, given that proactive employees are more likely to seek out new ideas and take initiative, digital leadership is more likely to provide greater empowerment to them. This will further contribute to their empowerment and lead them to participate in innovative behavior, attempting to maintain a balanced and equitable social exchange relationship. Thus, the following hypotheses are proposed:

**Hypothesis** **6a** **(H6a).** *A proactive personality has a significantly positive moderating effect on the relationship between digital leadership and psychological empowerment.*

**Hypothesis** **6b** **(H6b).** *A proactive personality has a positive moderating effect on the mediating effect of psychological empowerment on the relationship between digital leadership and employee innovative behavior.*

#### 2.5.2. The Moderating Role of a Proactive Personality in the Relationship between Digital Leadership, Affective Commitment, and Employee Innovative Behavior

Given that digital leadership has been discussed in the context of promoting affective commitment through three aspects, we believe that this influence is also influenced by a proactive personality. First, in terms of positive-framing-related aspects, positive framing by employees can evoke favorable associations and is more valuable and readily accepted [146]. Employees with a positive mindset can reframe their environment in a way that matches their understanding and are more likely to respect and be receptive to organizational values and culture [147]. Positive framing enables employees to recognize the positive effects of their actions, thereby motivating them to invest more energy in tasks [148]. Positive framing also allows employees to reappraise emotional cues in a more positive way, which will generate more positive feelings [149]. Second, in terms of sense-making-related aspects, employees’ affective commitment is subject to leaders’ social influence, which is associated not only with direct persuasion from leaders but also with employees’ sense-making in response to social cues provided by the leaders. Digital transformation-related change can be considered an emotional episode [150]; employees perceive change as events occur and organizational members interact over time. Third, in terms of relationship-building-related aspects, the more time and effort employees invest, the more emotional attachment they have. Employees who establish more relationships with leaders at work are more likely to gain shared values, beliefs, and social norms within the organization, which helps them cultivate a sense of belonging within the organization [151]. High-quality work relationships between leaders and colleagues can also further become a powerful source of connection, engagement, and vitality [152], thereby improving employees’ social resources and helping them integrate into their organization [153]. Thus, proactive employees are more likely to be influenced by digital leadership, and their affective commitment will be reinforced.

Furthermore, from the leaders’ perspective, given that proactive employees are more committed to work goals and put in higher levels of effort, digital leadership should be more likely to help provide support to employees in their work. This will further contribute to the development of high-quality commitments and enable employees to reciprocate by engaging in innovative behaviors that exceed formal expectations. Thus, the following hypotheses are proposed:

**Hypothesis** **7a** **(H7a).** *A proactive personality has a significantly positive moderating effect on the relationship between digital leadership and affective commitment.*

**Hypothesis** **7b** **(H7b).** *A proactive personality has a positive moderating effect on the mediating effect of affective commitment on the relationship between digital leadership and employee innovative behavior.*

**Figure 1:**
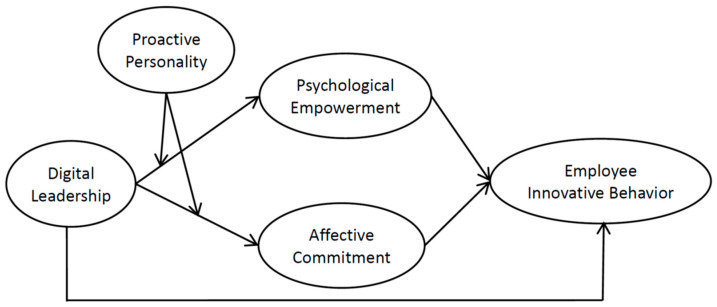
Research model.

Figure 1 illustrates our research model and summarizes the hypotheses.

## 3. Methodology

### 3.1. Measures

This study adopted scales developed in previous studies to design a Chinese version of the questionnaire. We employed the standard translation and back-translation procedures to translate the items from English into Chinese [154]. All constructs were rated on a five-point Likert scale (from 1 = “strongly disagree” to 5 = “strongly agree”).

#### 3.1.1. Independent Variable

Digital leadership (DL) as an independent variable was measured using six items adapted from a previous study [155]. One example item is “My leader is a digital expert”.

#### 3.1.2. Dependent Variable

Employee innovative behavior (EIB) as a dependent variable was measured with a six-item scale from a previous study [156]. One example item is “I search out new technologies, processes, techniques, and/or product ideas”.

#### 3.1.3. Mediating Variables

Psychological empowerment (PE) was measured on a twelve-item scale from a previous study [157]. One example item is “The work I do is very important to me”. Affective commitment (AC) was measured with a six-item scale used by a previous study [158]. One example item is “I Would be very happy to spend the rest of my career with my company”.

#### 3.1.4. Moderating Variable

Proactive personality (PP) was rated by using ten items used by a previous study [159]. One example item is “I am constantly on the lookout for new ways to improve my life”.

#### 3.1.5. Control Variables

Previous studies have suggested that it is necessary to control for competing correlated leadership variables to better test the effect of a focal leadership variable on outcomes [160]. Prior research has shown that transformational leadership can promote employee innovative behavior [161]. Therefore, we controlled for transformational leadership to examine the incremental effect of digital leadership on employee innovative behavior. Transformational leadership (TL) was measured by seven items of the Global Transformational Leadership (GTL) scale used by previous studies [162]. One example item is “My leader treats staff as individuals, supports and encourages their development”.

Furthermore, consistent with prior research [163], we also controlled for several demographic variables that might influence employee innovative behavior. We controlled for the gender variable (male = 0, female = 1), age variable (35 years old or below = 0, older than 35 years old = 1), tenure variable (less than 3 = 0, 3 years or more = 1), and education variable (college diploma or below = 0, bachelor’s degree or above = 1). We also controlled for industry variables (manufacturing = 0, other industry = 1).

### 3.2. Sample and Design

Following a cross-sectional study design together with the use of self-reports, we collected data from employees. The choice of a cross-sectional survey design was mainly due to the following two reasons. First, cross-sectional designs often become more informative when hypotheses are developed based on a strong and sound theory [164]. Second, the current study aims to examine the mediating role of psychological states, and it is difficult to predict how long digital leadership will take to affect each psychological state, which in turn influences employee innovative behavior. In this case, using a time-lagged design may pose a risk of erroneous conclusions about the strength of the relationships [165].

The questionnaire consisted of two parts: the first part was mainly the measurement items of the variables, and the second part was the demographic information of the respondents. The questionnaire was distributed in two stages, namely the pilot study and the main study.

A pilot study is considered essential for empirical studies with scale adaptation across different contexts [166]. Thus, before conducting the main study, a pilot study was performed to investigate the reliability and validity of the scales. The data collection process for the pilot survey adopted the same standards as for the main study.

Before conducting the pilot study, we contacted the human resource managers from five manufacturing firms that are implementing digital transformation in the Shandong province of China and then asked whether their firms were willing to participate in this survey. After obtaining approval from two of the firms, each manager introduced an employee from the human resource management department to assist in the survey. To carry out the work efficiently, two research assistants were employed to distribute and collect questionnaires within the firms. Before filling out the questionnaire, all participants were informed about the purpose of the study, that participation is entirely voluntary, and that personal information would be strictly protected. The participants were required to complete the questionnaire within 15 min. Upon completing the questionnaire, the participants sealed it in an envelope and returned it to the assistant. The pilot study was conducted in November 2022 and lasted for a week. In the pilot study, 175 data points were collected with 129 usable questionnaires (a response rate of approximately 73.71%). As a result of the pilot test, considering that the value of “Cronbach’s Alpha if Item Deleted” was higher than the value of “Cronbach’s alpha”, we deleted one item of digital leadership and one item of transformational leadership to make items fit in a scale [167]. Moreover, one item of employee innovative behavior, one item of affective commitment, and three items of proactive personality were deleted because factor loading was below 0.60. The final questionnaire contained a total of 40 items.

After the completion of the pilot survey implementation, individual items in the questionnaire were reasonably deleted and adjusted, and then the formal survey was carried out. To strengthen the explanation power, the formal survey expanded the sample to include employees from firms in different industries. These firms have adopted some digital technologies to support and improve their business and operational activities so that employees are sensitive to digital transformation and management issues. The participants were invited to fill out an electronic questionnaire through a link to an online survey. Participants were recruited in two ways. Firstly, the link to the survey was sent to friends and relatives and then forwarded to relevant employees they knew. Secondly, we indirectly sent questionnaires with the help of some MBA students who worked in the human resources department. In the web-based survey, informed consent for an anonymous survey was posted on social media first to recruit participants. Before filling out the online questionnaire, all participants were also informed of the purpose of the study, that participation is entirely voluntary, and that personal information would be strictly protected. All participants were sent an online link to the questionnaire and were required to complete it. To ensure the quality of the online questionnaire, we added screening criteria questions to the questionnaire to ensure that the respondents’ identities met the requirements of this study. In addition, we set a time limit for answering the questionnaire, and questionnaires that were completed in less than 5 min or more than 8 h were considered invalid. The formal survey was conducted from 15 January to 8 April 2023. A total of 509 questionnaires were collected in two ways. After removing 150 invalid questionnaires due to reasons such as careless responses and obvious contradictions in the answers to positive and negative questions, there were 359 remaining valid questionnaires, with a valid data recovery rate of 70.53%. Moreover, the independent sample *T*-test was used to analyze the difference in the sample data collected in the two ways. The results indicated there was no significant difference between the two-sample data, which could be combined into one dataset for the following analysis. The demographic characteristics of the study respondents are shown in Table 1.

## 4. Results

All statistical analyses were conducted using SPSS 27.0, AMOS 27.0, and SmartPLS 3.2.9. SEM was run with AMOS 27.0 and SmartPLS 3.2.9 to test reliability, validity, and model fit. To examine the hypotheses, this study used a partial least-squares (PLS) approach through SmartPLS 3.2.9.

### 4.1. Reliability and Validity

This study used a three-step method to check the reliability and validity of the measures. First, we calculated Cronbach’s alpha to test the reliability. The Cronbach’s alpha value of each construct ranged from 0.905 to 0.963 (see Table 2), which was greater than the recommended threshold value of 0.70, indicating adequate reliability [168].

Second, we conducted an exploratory factor analysis (EFA) to check the unidimensionality of the operationalized measures. A principal component analysis (PCA) was chosen for all measurement items, while Varimax rotation with Kaiser normalization was used to clarify the factors in the exploratory factor analysis. The results showed that six factors with eigenvalues above 1.0 emerged, explaining 74.38% of the total variance. In addition, the KMO value of all the constructs was 0.958, confirming that the data were suitable for the factor analysis.

Third, we employed a confirmatory factor analysis (CFA) to examine whether the data fit our hypothesized measurement model and to assess the validity of the measures. We analyzed the values of χ^2^, χ^2^/df, GFI, AGFI, RMR, RMSEA, and CFI to check the fit indexes (see Table 3), which revealed that the measurement model achieved a satisfactory level of fit. As shown in Table 2, all the factor loadings of the items were higher than 0.70. The values of composite reliabilities (CRs) and the values of the average variance extracted (AVE) showed acceptable values (0.905~0.963, 0.656~0.765, respectively) above the required thresholds (0.70 and 0.50 accordingly), providing support for convergent validity [168]. For discriminant validity, we compared the fitting results of the one-factor, two-factor, three-factor, four-factor, and five-factor models (see Table 4). According to the results in Table 4, each index of the five-factor model was significantly better than the other models, indicating that the five core variables in this study have good discriminant validity [169].

Furthermore, the Fornell–Larcker criterion and the heterotrait–monotrait ratio of correlations (HTMT) criteria were employed to test the discriminant validity. The results are shown in Table 5 and Table 6. The results in Table 5 and Table 6 met the required level, indicating a clear differentiation in validity across the constructs.

### 4.2. Common Method Variance

Because our measures of variables were reported by employees themselves, we also checked for common method variance (CMV) using two approaches, namely Harman’s single factor and the variance inflation factor (VIF). Regarding the first approach, the findings demonstrated that the first factor can explain 38.86% of variances less than 50%. This suggests that the CMV of the main variables was not significant [170]. Concerning the second approach, the VIF values ranged between 1.075 and 2.407, meaning that CMV and multicollinearity are not a concern for this study [171].

### 4.3. Descriptive Statistics and Correlation Analysis

The descriptive statistics and correlations of the variables are displayed in Table 7. According to the results in Table 7, there are no cases where the standard deviation is greater than the mean, and the stability of the sample data is good. The difference between the maximum and minimum values of the main variables is significant, indicating that there are significant differences. It can also be seen from the statistical results that the skewness of all the sample data is between −2 and 2, and the kurtosis is between −3 and 3. The values of skewness and kurtosis are within an acceptable range, which indicates that the data distribution conforms to the characteristics of a Gaussian distribution. Moreover, digital leadership is positively related to employee innovative behavior (r = 0.477, *p* < 0.001), psychological empowerment (r = 0.546, *p* < 0.001), and affective commitment (r = 0.422, *p* < 0.001). Furthermore, psychological empowerment and affective commitment are positively related to employee innovative behavior (r = 0.517, *p* < 0.001 and r = 0.419, *p* < 0.001, respectively), and proactive personality is positively related to employee innovative behavior (r = 0.213, *p* < 0.001). These results are consistent with our assumptions. Given that the influence of many of the control variables is minimal, only transformational leadership is retained in the model to test the hypotheses.

### 4.4. Hypothesis Testing: Direct Effect and Mediation Effects

**Figure 2:**
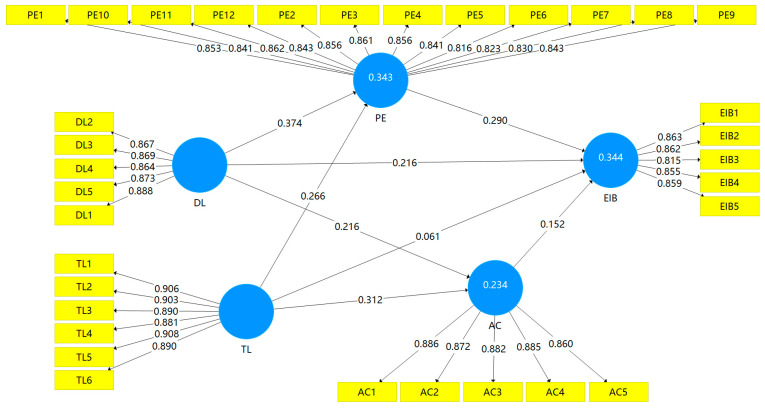
A structural model with mediation effects.

To examine the direct effect and mediation effects, bootstrapping was carried out using SmartPLS 3.2.9 with 5000 subsamples based upon percentile bootstrapping with a two-tailed test type and a significance level of 0.05. Figure 2 and Table 8 portray the results of the structural path analysis. The fit indexes of the model are satisfactory (SRMR = 0.033, d_ULS = 0.610, d_G = 0.367, NFI = 0.930, RMS_theta = 0.095), suggesting that the model was reasonably well fitted in general in this research. The results given in Figure 2 show that the outer loading of the indicators of the reflective constructs is above the cut-off value of 0.70, and the reliability of the study constructs is established. Figure 2 and Table 8 also show R^2^ values of 0.343, 0.234, and 0.344 for psychological empowerment, affective commitment, and employee innovative behavior, respectively. Moreover, the Q^2^ of the model was determined using a cross-validation redundancy approach, and Q^2^ values of 0.24, 0.176, and 0.245 for psychological empowerment, affective commitment, and employee innovative behavior were obtained, respectively.

H1 predicted that digital leadership is positively related to employee innovative behavior. The total effect given in Table 8 shows a strong and significant positive relationship between digital leadership and employee innovative behavior (β = 0.357, *p* = 0.000, 95% CI = 0.214, 0.492). Hence, H1 is accepted.

H2, H3, H4, and H5 proposed that psychological empowerment and affective commitment mediate the relationship between digital leadership and employee innovative behavior. The results in Table 8 show that digital leadership has a positive and significant effect on psychological empowerment (β = 0.374, *p* = 0.000, 95% CI = 0.247, 0.491) and affective commitment (β = 0.216, *p* = 0.001, 95% CI = 0.083, 0.343). Hence, H2 and H4 are accepted. Although the results show that psychological empowerment significantly mediates the relationship between digital leadership and employee innovative behavior (β = 0.108, *p* = 0.001, 95% CI = 0.054, 0.186), affective commitment does not mediate the relationship between digital leadership and employee innovative behavior (β = 0.033, *p* = 0.070, 95% CI = 0.006, 0.078). Moreover, the influence of digital leadership on employee innovative behavior remains significant with the introduction of mediating effects (β = 0.216, *p* = 0.002, 95% CI = 0.077, 0.358). Although with the introduction of psychological empowerment and affective commitment, the β-value of the effect of digital leadership on employee innovative behavior decreases from 0.357 to 0.216, the mediation effects are found to be significant (β = 0.141, *p* = 0.000, 95% CI = 0.083, 0.213). Hence, H3 is accepted, and H5 is not supported. Psychological empowerment partially mediates the relationship between digital leadership and employee innovative behavior.

### 4.5. Hypothesis Testing: Moderating Effects

**Figure 3:**
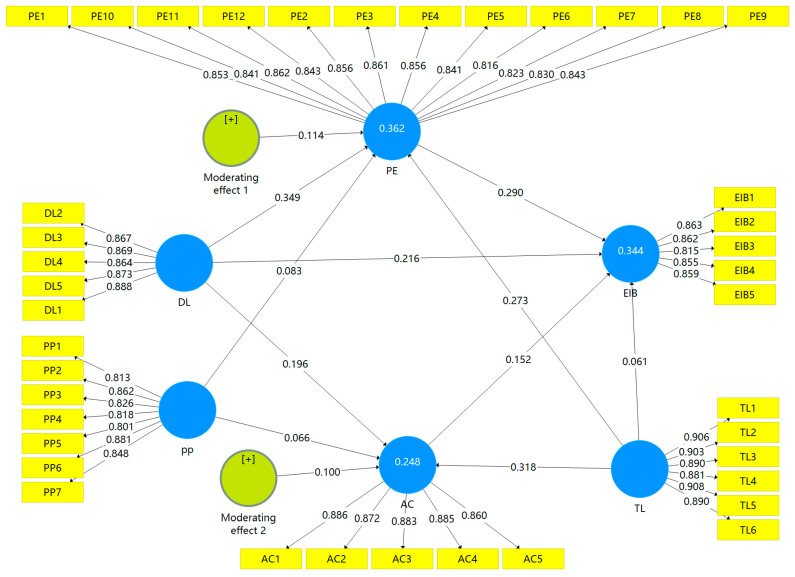
A structural model with moderating effects.

To examine the moderating effects of a proactive personality, bootstrapping was carried out using SmartPLS 3.2.9 with 5000 subsamples based upon percentile bootstrapping with a two-tailed test type and a significance level of 0.05. Figure 3 and Table 9 present the results of the structural path analysis. The fit indexes of the model are satisfactory (SRMR = 0.035, d_ULS = 0.944, d_G = 0.521, NFI = 0.917, RMS_theta = 0.107), suggesting that the model was reasonably well fitted in general in this research. The results given in Figure 3 show that the outer loading of the indicators of the reflective constructs is above the cut-off value of 0.70, and the reliability of the study constructs is established. Figure 3 also shows R^2^ values of 0.362, 0.248, and 0.344 for psychological empowerment, affective commitment, and employee innovative behavior, respectively. These R^2^ values establish the good predictive accuracy of the research model.

H6a and H7a proposed that a proactive personality moderates the direct effect of digital leadership on psychological empowerment and affective commitment, while H6b and H7b proposed that a proactive personality moderates the indirect effect of digital leadership on employee innovative behavior through psychological empowerment but not affective commitment. The interaction approach was used to calculate the moderating effects.

According to the results in Table 9, the interaction effect of digital leadership and a proactive personality on psychological empowerment is positive but not significant (β = 0.114, *p* = 0.093, 95% CI = −0.219, 0.188), and the interaction effect of digital leadership and a proactive personality on affective commitment is also positive but not significant (β = 0.100, *p* = 0.095, 95% CI = −0.215, 0.162). Thus, a proactive personality neither moderates the direct relationship between digital leadership and psychological empowerment nor the direct relationship between digital leadership and affective commitment. Therefore, H6a and H7a are not supported.

Moreover, the interaction effect of digital leadership and a proactive personality on employee innovative behavior through psychological empowerment is positive but not significant (β = 0.033, *p* = 0.129, 95% CI = −0.058, 0.063), and the interaction effect of digital leadership and a proactive personality on affective commitment is also positive but not significant (β = 0.015, *p* = 0.207, 95% CI = −0.026, 0.034). These results indicate that a proactive personality may not be able to moderate the indirect effect of digital leadership on employee innovative behavior.

**Figure 4:**
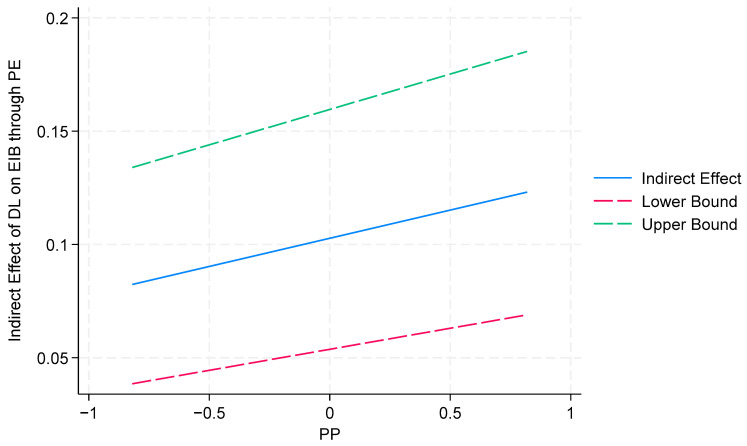
The conditional effect of a PP on the indirect effect of DL on EIB through PE.

**Figure 5:**
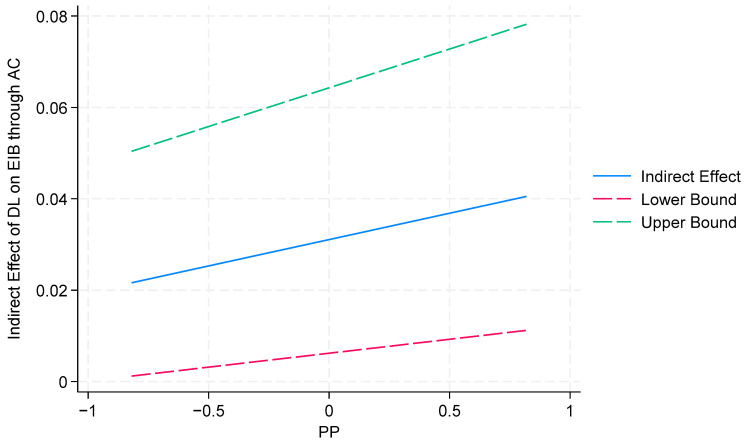
The conditional effect of a PP on the indirect effect of DL on EIB through AC.

Furthermore, to better illustrate the mediated–moderated effects of a proactive personality, a condition mediation analysis in SEM was employed [172]. The indirect effect estimates at varying levels of proactive personality are shown in Table 10. The study probed the interactions via a simple slope analysis and used Stata 18 to present the results (shown in Figure 4 and Figure 5). According to the results in Figure 4 and Figure 5, the slopes of the three straight lines are almost parallel, indicating that a proactive personality does not significantly positively moderate the indirect effect of digital leadership on employee innovative behavior through psychological empowerment and affective commitment. Thus, H6b and H7b are not supported.

### 4.6. Robustness Analysis

To assess the robustness of the SEM, this study used SmartPLS 3.2.9 to conduct a multi-group analysis to test the moderating effect of a proactive personality. A proactive personality was classified into categorical variables using the average value. Employees in Group 1 had a low level of proactive personality (n = 153), and those in Group 2 had a high level of proactive personality (n = 206). The detailed results are presented in Table 11. Although the effects of the high-level group are generally better than those of the low-level group in Table 11, the *p* values show that there is no significant difference between these two groups. There is no significant difference between the different groups. Thus, a proactive personality does not moderate the direct effect of digital leadership on psychological empowerment and affective commitment or the indirect effect of digital leadership on employee innovative behavior. These results are consistent with those of the structural equation model.

### 4.7. Endogeneity Analysis

An instrumental variable two-stage least-squares (IV-2SLS) regression was used to analyze the endogeneity issue regarding the relationship between digital leadership and employee innovative behavior. Instrumental variables are typically required to be highly correlated with the independent variable and not affect the outcome variable through other paths. The mean of digital leadership in the same industry (MDLH) was chosen as an instrumental variable. On the one hand, digital leadership in the same industry can involve mutual learning, dissemination, and influence among managers. However, the digital leadership of a single firm cannot easily influence the digital leadership of the entire industry. On the other hand, individual employees’ innovative behavior is usually not related to the average digital leadership in the same industry but is more influenced by the digital leadership of their firm.

To address endogeneity with the IV regression, the EndoS macro for SPSS was employed [173]. EndoS conducts a two-stage ordinary least-squares (OLS) regression using residuals as the independent variables and generates a joint F-test incorporating multiple endogenous variables. Table 12 gives estimations from the OLS and IV-2SLS regressions.

Comparing the results of the OLS and IV-2SLS regressions in Table 12, it was found that the parameter estimates on digital leadership for employee innovative behavior increased in the IV-2SLS estimates, and this was also statistically significant. Hausman’s specification test was used to check whether OLS regression is efficient. The F value is 2.041 (*p* = 0.154), which shows that digital leadership is exogenous. The over-identifying restriction test is used to test whether instrumental variables are exogenous. The results of the J-statistic show that the null hypothesis is not rejected at the 5% significance level, indicating that the MDLH is indeed valid. The Cragg–Donald F statistic is used to test whether instrumental variables are weak. The Cragg–Donald F statistic (22.74) exceeds the critical value of the Stock–Yogo test at the level of 10% 2SLS size (critical value is 16.38). Thus, the MDLH is not a weak instrument. These results indicate that endogeneity was likely not influential in the relationship between digital leadership and employee innovative behavior.

## 5. Discussion and Implications

### 5.1. Discussion

Building on the cognitive–affective processing framework, this study explores the underlying mechanisms and boundary conditions that explain why and under what circumstances digital leadership relates to employee innovative behavior. The major findings are discussed as follows.

Firstly, the results show that digital leadership can directly promote employee innovative behavior, as we expected. Previous studies have mainly explored the effect of digital leadership on team and organizational performance [47,48], but empirical investigations concerning the individual outcomes of digital leadership need further development. Our research empirically indicates that employees can obtain innovative benefits using digital leadership. The findings are consistent with previous research [20,21]. Thus, this study offers novel insights into the study of digital leadership.

Secondly, to better understand how digital leadership affects employee innovative behavior, we conducted further research to explore the underlying mechanisms that link digital leadership and employee innovative behavior. Drawing on the cognitive–affective processing framework, the findings reveal that psychological empowerment partially mediates the relationship between digital leadership and employee innovative behavior, indicating that psychological empowerment is an important mechanism linking digital leadership and employee innovative behavior. This study reveals that digital leadership could establish and strengthen employees’ perceptions of psychological empowerment, thus generating a positive association with employee innovative behavior. Thus, this study validates the critical role that leaders play in promoting psychological empowerment, which is consistent with the previous studies [174]. Contrary to our expectations, affective commitment does not mediate the relationship between digital leadership and employee behavior. However, this effect is only not significant at the 5% level (β = 0.033, *p* = 0.070). We suspect that this result is caused by omitting some items with low factor loadings, which decreases the statistical power. Therefore, if all items were loaded well, we may have found a significant effect. Although this finding stands in contrast to previous research that reported affective commitment to mediate the relationship between leadership style and employees’ outcomes [175], it cannot be denied that affective commitment has a potential mediating role. In fact, previous studies have also found that affective commitment did not mediate the relationship between transformational leadership and innovative work behavior [176].

Thirdly, this research explores the boundary conditions of the effect of digital leadership on employee innovative behavior. Contrary to what was hypothesized, we found that a proactive personality cannot significantly moderate the direct effect of digital leadership on psychological empowerment and affective commitment or the indirect effect of digital leadership on employee innovative behavior. However, the interaction effect of digital leadership and a proactive personality on psychological empowerment is only not significant at the 5% level (β = 0.114, *p* = 0.093), and the interaction effect of digital leadership and a proactive personality on affective commitment is also only not significant at the 5% level (β = 0.100, *p* = 0.095). Therefore, based on these results, we cannot deny the importance of a proactive personality. Moreover, based on the CAPS theory, one potential explanation could be that the contextual cues of digital leadership may not have a sufficient connection to a proactive personality. Specifically, a proactive personality may have a positive impact on individuals viewing digital transformation as an opportunity while having a negative effect on individuals viewing digital transformation as a threat. Moreover, a substitute for leadership theory may provide another perspective to explain this finding. Previous studies have found that positive follower traits can replace leadership styles and weaken the connection between leadership and outcomes [177]. Thus, employees with a high level of proactive personality have higher levels of psychological empowerment and affective commitment, irrespective of digital leadership. On the contrary, employees lacking a proactive personality benefit more from the effects of digital leadership.

### 5.2. Theoretical Contributions

The findings of this study can contribute to the existing literature on digital leadership and employee innovative behavior in several ways.

Firstly, our research contributes to leadership theory by building on the discussion of the implications of digital leadership. Previous research has demonstrated that different leadership styles have effects on employee innovative behavior [10,11,13,14]. However, as an important leadership style [30,37], the consequences of digital leadership have not yet been explored. Our research provides empirical evidence of the beneficial effects of digital leadership on individual outcomes, especially employee innovative behavior. Moreover, we empirically respond to the call for studying the effects of digital leadership on different outcomes [49,50].

Secondly, our study explores the complex cognitive and affective mechanisms of the effect of digital leadership on employee innovative behavior through the dual mediating roles of psychological empowerment and affective commitment. Previous studies have discussed the relationship between leadership style and employee innovative behavior based on the theories of social learning [178] and self-determination [179], which stem from a single path of cognition or affect. According to the CAPS, individual behavior is a combination of cognition and affect, and only focusing on a single path is not enough to explain the complex mechanism of employee innovative behavior. Although the mediating role of affective commitment is not significant, it cannot be denied that affective factors may play an important mediating role. Therefore, drawing on the CAPS, our study provides a new perspective from a dual mechanism for the research of digital leadership and employee innovative behavior. This analytical approach is also consistent with previous research that has employed the CAPS perspective to analyze the relationship between leadership style and employee outcomes [180]. Furthermore, we have also empirically responded to the call for more research into the impact mechanisms of digital leadership [20].

Thirdly, our research attempts to combine CAPS theory with personality theory to address the shortcomings of the CAPS in explaining how digital leadership affects employee cognitive–affective units and subsequent innovative behavior. Although the moderating effects of a proactive personality are not significant, they are still positive. In fact, previous studies have shown that a proactive personality can have both positive effects on the effectiveness of leadership style [181] and may not positively moderate the indirect relationship between leadership style and employee behavior [182]. Thus, the present research provides a contingency view of the innovative implications of digital leadership and responds to the research call for investigations of the extent to which digital leadership is effective [18,85].

### 5.3. Managerial Implications

Nowadays, digitalization has become an unstoppable and irreversible trend for various industries and firms. The digital economy has played an important role in promoting firms’ rapid development. Thus, the findings of this study may have important implications for managers in the context of digital transformation.

Firstly, managers should embrace digital leadership philosophy in their firm agendas. To better respond to the needs of digital transformation, many firms are focusing on the development of leadership and have established the position of Chief Digital Officers to drive their firms’ functional transformation. Considering that employee innovation is the foundation of organizational innovation and digital leadership can effectively promote employee innovative behavior, managers need to pay attention to digital leadership and establish an awareness of valuing digital leadership. For example, the core firm of TSINGHUA UNIGROUP, H3C, has developed a brand new digital leadership model from cognition and behavior to culture, including digital thinking architecture, digital strategy execution, and digital corporate culture construction.

Secondly, staffing policies in firms should consider hiring leaders who can discuss and address digital issues and may consider training and coaching their leaders to deal with digital initiatives. To achieve each component of digital leadership, managers should work together with leaders and assist them in acquiring the required digital abilities and mindsets. Specifically, managers should assist leaders in explaining why digital transformation practices are vital and how leaders can help firms achieve such practices. Moreover, managers may establish scientific and reasonable processes to select and promote leaders with digital abilities, carry out training plans and projects to advocate for leaders’ digital abilities, and formulate performance evaluation systems to encourage the development of leaders’ digital abilities. In the presence of such characteristics and abilities in a leader, employees are more likely to exhibit innovative behavior. For example, PwC’s unique Digital Leadership Program can provide firms with services such as digital leadership research and diagnosis, digital leadership training, and improvement courses. The PwC digital leadership model involves six aspects: top-level thinking, digital intelligence, scenario breakthroughs, digital organizing, subverting conventions, and digital ethics.

Thirdly, managers should highlight the importance of employees’ psychological empowerment, as it fosters employee innovative behavior. Generating new ideas is a trial-and-error process, so digital leadership should build a supportive context to encourage employees to take risks. Therefore, managers should create a vision for innovation by recognizing employees’ innovative work, providing employees with autonomy in work-related activities, helping employees clarify their roles more clearly, and tolerating employees for making mistakes and failing to achieve expected goals. For example, Deloitte offers the Greenhouse Innovation Laboratory, which provides an immersive innovation experience, comprehensive visual and sensory activation, flexible scene settings, and high-tech support, allowing employees to work together in a flatter and more harmonious manner, inspiring inspiration and innovative ideas.

### 5.4. Limitations and Future Research

This study has some limitations that can be addressed by future studies. First, based on the cognitive–affective processing framework, this study examined the effects of two mechanisms of digital leadership on employee innovative behavior. This research method is closely related to previous research [180], which helps to comprehensively reveal the impact of digital leadership from multiple perspectives. Thus, future research can consider other mechanisms from various theoretical perspectives, such as social exchange theory, social learning theory, and social identity theory.

Secondly, although this study investigated the moderating role of a proactive personality, existing studies have suggested that, in addition to individual traits, work characteristics, leader–member relationships, and leaders’ characteristics also affect the effectiveness of leadership [183]. Future research could explore other potential moderators, such as task interdependence, power distance, or leader–member exchange.

Lastly, the measurements of the main variables were self-reported by employees, which may lead to them being overestimated or underestimated. In the future, it will be possible to use multi-source data from employees and leaders to obtain more objective data and provide additional insights.

## 6. Conclusions

Building on the cognitive–affective processing framework, the present research found a positive link between digital leadership and employee innovative behavior; this relationship was mediated by psychological empowerment. Moreover, the mediating role of affective commitment and the moderating effects of a proactive personality were positive but not significant. These findings provide inspiring insights regarding how to use digital leadership to promote employee innovative behavior.

## Figures and Tables

**Table 1:** Sample demographic characteristics (n = 359).

Characteristic	Classification	Frequency	%
Gender	Male	155	43.18%
	Female	204	56.82%
Age	25 years old or below	68	18.94%
	26–35	120	33.43%
	36–45	97	27.02%
	46 years old or above	74	20.61%
Education	High school or below	45	12.53%
	College diploma	102	28.41%
	Bachelor’s degree	174	48.47%
	Master’s degree or above	38	10.58%
Tenure	3 years or below	165	45.96%
	4–10 years	103	28.69%
	11 years or above	91	25.35%
Industry	Manufacturing	89	24.79%
	IT	51	14.21%
	Retail trade	38	10.58%
	Finance	20	5.57%
	Education and training	57	15.88%
	Real estate/construction	27	7.52%
	Business services	25	6.96%
	Other	52	14.48%

**Table 2:** Analysis of the construct measures’ validity and reliability (n = 359).

Construct and Its Items	Factor Loading
Digital Leadership (Cronbach’s alpha = 0.922; AVE = 0.701; CR = 0.922)	
DL1: My leader is a digital expert	0.708
DL2: When it comes to digital knowledge, my leader is always up-to-date	0.757
DL3: My leader driving the digital transformation forward proactively in our unit	0.715
DL4: My leader can make others enthusiastic about the digital transformation	0.778
DL5: My leader has a clear idea of the structures and processes that are needed for the digital transformation	0.759
Employee Innovative Behavior (Cronbach’s alpha = 0.905; AVE = 0.656; CR = 0.905)	
EIB1: I search out new technologies, processes, techniques, and/or product ideas	0.768
EIB2: I often come up with innovative ideas	0.766
EIB3: I do my best to get the resources he/she needs to realize innovative ideas	0.782
EIB4: I make appropriate plans to implement new ideas	0.761
EIB5: On the whole, I am an innovative person	0.784
Psychological Empowerment (Cronbach’s alpha = 0.963; AVE = 0.686; CR = 0.963)	
PE1: The work I do is very important to me	0.796
PE2: My job activities are personally meaningful to me	0.809
PE3: The work I do is meaningful to me	0.803
PE4: I am confident about my ability to do my job	0.784
PE5: I am self-assured about my capabilities to perform my work activities	0.782
PE6: I have mastered the skills necessary for my job	0.766
PE7: I have significant autonomy in determining how I do my job	0.761
PE8: I can decide on my own how to go about doing my work	0.764
PE9: I have considerable opportunity for independence and freedom in how I do my job	0.778
PE10: My impact on what happens in my department is large	0.787
PE11: I have a great deal of control over what happens in my department	0.826
PE12: I have a significant influence over what happens in my department	0.797
Affective Commitment (Cronbach’s alpha = 0.925; AVE = 0.713; CR = 0.925)	
AC1: I Would be very happy to spend the rest of my career with my company	0.806
AC2: I really feel as if my company’s problems are my own	0.825
AC3: I feel a strong sense of “belonging” to my company	0.810
AC4: I feel emotionally attached to my company	0.795
AC5: I feel like “part of the family” at my company	0.782
Proactive Personality (Cronbach’s alpha = 0.930; AVE = 0.656; CR = 0.930)	
PP1: I am constantly on the lookout for new ways to improve my life	0.831
PP2: Wherever I have been, I have been a powerful force for constructive change	0.841
PP3: Nothing is more exciting than seeing my ideas turn into reality	0.835
PP4: No matter what the odds, if I believe in something I will make it happen	0.828
PP5: I excel at identifying opportunities	0.836
PP6: If I believe in an idea, no obstacle will prevent me from making it happen	0.845
PP7: I can spot a good opportunity long before others can	0.834
Transformational Leadership (Cronbach’s alpha = 0.951; AVE = 0.765; CR = 0.951)	
TL1: My leader treats staff as individuals, supports and encourages their development	
TL2: My leader gives encouragement and recognition to staff	0.821
TL3: My leader fosters trust, involvement, and cooperation among team members	0.798
TL4: My leader encourages thinking about problems in new ways and questions assumptions	0.794
TL5: My leader is clear about his/her values and practices what he/her preaches	0.825
TL6: My leader instills pride and respect in others and inspires me by being highly competent	0.826

**Table 3:** Fit indexes of the variables’ measurement models (n = 359).

Model	χ^2^	χ^2^/df	GFI	AGFI	RMR	RMSEA	CFI
Digital Leadership	9.306	1.861	0.990	0.970	0.013	0.049	0.997
Employee Innovative Behavior	8.722	1.744	0.990	0.971	0.012	0.046	0.997
Psychological Empowerment	78.906	1.461	0.965	0.949	0.012	0.036	0.993
Affective Commitment	7.845	1.569	0.992	0.975	0.010	0.040	0.998
Proactive Personality	22.121	1.580	0.983	0.966	0.015	0.040	0.995
Transformational Leadership	16.362	1.818	0.985	0.964	0.010	0.048	0.996

**Table 4:** Results of discriminant validity (n = 359).

Model	Factor	χ^2^	df	χ^2^/df	CFI	TLI	SRMR	RMSEA
Five-factor model	DL + PE + AC + PP + EIB	637.222	517	1.233	0.988	0.986	0.031	0.025
Four-factor model	DL; PE; AC; PP + EIB	1789.176	521	3.434	0.869	0.859	0.165	0.082
Three-factor model	DL; PE; AC + PP + EIB	3204.184	524	6.115	0.722	0.703	0.142	0.120
Three-factor model	DL; AC; PE + PP + EIB	3122.314	524	5.959	0.731	0.712	0.140	0.118
Two-factor model	DL; PE + AC + PP + EIB	4051.188	526	7.702	0.635	0.611	0.151	0.137
One-factor model	DL + PE + AC + PP + EIB	4865.386	527	9.232	0.551	0.522	0.158	0.152

Note: DL = digital leadership; PE = psychological empowerment; AC = Affective commitment; PP = proactive personality; EIB = employee innovative behavior.

**Table 5:** Fornell–Larcker criterion (n = 359).

Variables	1	2	3	4	5	6
1. Employee Innovative Behavior	0.851					
2. Digital Leadership	0.481	0.872				
3. Psychological Empowerment	0.520	0.551	0.844			
4. Affective Commitment	0.423	0.424	0.524	0.877		
5. Proactive Personality	0.224	0.667	0.108	0.082	0.836	
6. Transformative Leadership	0.423	0.667	0.515	0.456	0.040	0.896

**Table 6:** Heterotrait–monotrait ratio (n = 359).

Variables	1	2	3	4	5	6
1. Employee Innovative Behavior						
2. Digital Leadership	0.524					
3. Psychological Empowerment	0.554	0.583				
4. Affective Commitment	0.460	0.457	0.555			
5. Proactive Personality	0.232	0.081	0.105	0.084		
6. Transformative Leadership	0.455	0.710	0.537	0.484	0.048	

**Table 7:** Mean, standard deviation, and correlations (n = 359).

Variables	1	2	3	4	5	6	7	8	9	10	11
1. EIB	1.000										
2. DL	0.477 ***	1.000									
3. PE	0.517 ***	0.546 ***	1.000								
4. AC	0.419 ***	0.422 ***	0.524 ***	1.000							
5. PP	0.213 ***	0.075	0.099	0.069	1.000						
6. TL	0.421 ***	0.665 ***	0.514 ***	0.455 ***	0.029	1.000					
7. Sex	0.042	−0.089	−0.007	0..072	−0.062	−0.008	1.000				
8. Age	0.004	−0.035	−0.053	0.000	−0.079	−0.132 *	−0.081	1.000			
9. Education	0.117 *	0.010	0.031	0.037	−0.099	0.074	0.029	−0.125 *	1.000		
10. Tenure	−0.131 *	−0.069	−0.096	−0.082	−0.065	−0.097	0.103	−0.211 ***	−0.201 ***	1.000	
11. Industry	−0.044	−0.019	0.007	−0.011	−0.039	−0.011	0.151 **	−0.008	0.125 *	−0.044	1.000
Mean	3.651	3.527	3.682	3.422	0.368	3.675	0.568	0.476	0.591	0.730	0.752
SD	0.798	0.889	0.719	0.859	0.819	0.879	0.496	0.500	0.492	0.445	0.432
Min	1.000	1.000	1.000	1.000	1.000	1.000	0.000	0.000	0.000	0.000	0.000
Max	5.000	5.000	5.000	5.000	5.000	5.000	1.000	1.000	1.000	1.000	1.000
Skewness	−0.733	−0.734	−0.887	−0.503	−0.548	−0.903	−0.277	0.095	−0.370	−1.039	−1.173
Kurtosis	0.360	0.067	0.626	0.147	0.227	0.585	−1.934	−2.002	−1.874	−0.925	−0.629

Note. * *p* < 0.05; ** *p* < 0.01; *** *p* < 0.001.

**Table 8:** Results of main and indirect effects.

Relationships	β	SD	T	*p*	95% CI-PC	95% CI-BC	VIF	f^2^	R^2^	Q^2^
Direct effects										
DL → EIB	0.216 ***	0.071	3.028	0.002	[0.076, 0.358]	[0.077, 0.358]	2.201	0.035	0.344	0.245
DL → PE	0.374 ***	0.062	6.004	0.000	[0.249, 0.493]	[0.247, 0.491]	1.799	0.118	0.343	0.240
DL → AC	0.216 ***	0.065	3.307	0.001	[0.084, 0.343]	[0.083, 0.343]	1.799	0.034	0.234	0.176
PE → EIB	0.290 ***	0.069	4.188	0.000	[0.154, 0.428]	[0.154, 0.427]	1.726	0.074		
AC → EIB	0.152 *	0.063	2.427	0.015	[0.030, 0.276]	[0.029, 0.275]	1.481	0.024		
Indirect effects										
DL → PE → EIB	0.108 ***	0.032	3.338	0.001	[0.050, 0.180]	[0.054, 0.186]				
DL → AC → EIB	0.033	0.018	1.815	0.070	[0.004, 0.075]	[0.006, 0.078]				
Total indirect effects										
DL → EIB	0.141 ***	0.033	4.303	0.000	[0.082, 0.211]	[0.083, 0.213]				
Total effect										
DL → EIB	0.357 ***	0.070	5.069	0.000	[0.216, 0.495]	[0.214, 0.492]				
Control variable										
TL → PE	0.266 ***	0.064	4.173	0.000	[0.145, 0.391]	[0.143, 0.389]	1.799	0.060		
TL → AC	0.312 ***	0.066	4.699	0.000	[0.183, 0.443]	[0.181, 0.440]	1.799	0.071		
TL → EIB (direct)	0.061	0.075	0.819	0.413	[−0.085, 0.208]	[−0.088, 0.205]	1.975	0.003		
TL → PE → EIB	0.077 **	0.027	2.855	0.004	[0.032, 0.136]	[0.035, 0.142]				
TL → AC → EIB	0.047 *	0.020	2.317	0.021	[0.011, 0.092]	[0.013, 0.095]				
TL → EIB (total indirect)	0.124 ***	0.032	3.924	0.000	[0.068, 0.194]	[0.070, 0.197]				
TL → EIB (total)	0.186 *	0.075	2.458	0.014	[0.038, 0.336]	[0.034, 0.333]				

Note. * *p* < 0.05; ** *p* < 0.01; *** *p* < 0.001.

**Table 9:** Results of moderating effects.

Relationships	β	SD	T	*p*	95% CI-PC	95% CI-BC	VIF	f^2^	R^2^	Q^2^
Direct effects										
DL → EIB	0.216 **	0.070	3.061	0.002	[0.072, 0.351]	[0.074, 0.353]	2.021	0.035	0.344	0.245
DL → PE	0.349 ***	0.061	5.735	0.000	[0.226, 0.462]	[0.231, 0.467]	1.832	0.104	0.362	0.253
DL → AC	0.196 **	0.065	3.011	0.003	[0.064, 0.316]	[0.067, 0.319]	1.831	0.028	0.248	0.185
PE → EIB	0.290 ***	0.067	4.300	0.000	[0.154, 0.422]	[0.153, 0.420]	1.726	0.074		
AC → EIB	0.152 *	0.061	2.477	0.013	[0.035, 0.274]	[0.029, 0.270]	1.481	0.024		
DL × P → PE	0.114	0.068	1.682	0.093	[−0.081, 0.232]	[−0.219, 0.188]	1.031	0.022		
DL × PP → AC	0.100	0.060	1.670	0.095	[−0.053, 0.208]	[−0.215, 0.162]	1.029	0.014		
PP → PE	0.083	0.053	1.563	0.118	[−0.031, 0.187]	[−0.068, 0.167]	1.026	0.011		
PP → AC	0.066	0.059	1.129	0.259	[−0.074, 0.167]	[−0.125, 0.144]	1.024	0.006		
Indirect effects										
DL → PE → EIB	0.101 ***	0.029	3.430	0.001	[0.047, 0.163]	[0.051, 0.170]				
DL → AC → EIB	0.030	0.017	1.789	0.074	[0.004, 0.068]	[0.006, 0.071]				
DL ×PP → PE→ EIB	0.033	0.022	1.519	0.129	[−0.024, 0.077]	[−0.058, 0.063]				
DL × PP → AC→ EIB	0.015	0.012	1.262	0.207	[−0.005 0.042]	[−0.026, 0.034]				
PP → PE→ EIB	0.024	0.018	1.309	0.191	[−0.008, 0.068]	[−0.013, 0.061]				
PP → AC→ EIB	0.010	0.011	0.894	0.371	[−0.010, 0.036]	[−0.015, 0.033]				
Total indirect effects										
DL → EIB	0.131 ***	0.030	4.359	0.000	[0.076, 0.193]	[0.080, 0.201]				
PP → EIB	0.034	0.023	1.491	0.136	[−0.016, 0.084]	[−0.034, 0.070]				
Total effect										
DL → EIB	0.347 ***	0.069	5.017	0.000	[0.205, 0.479]	[0.207, 0.484]				
Control variable										
TL → PE	0.273 ***	0.062	4.441	0.000	[0.153, 0.392]	[0.155, 0.394]	1.802	0.065		
TL → AC	0.318 ***	0.066	4.818	0.000	[0.188, 0.446]	[0.191, 0.450]	1.802	0.075		
TL → EIB (direct)	0.061	0.073	0.833	0.405	[−0.085, 0.203]	[−0.089, 0.197]	1.974	0.003		
TL → PE → EIB	0.079 **	0.027	2.927	0.003	[0.033, 0.138]	[0.036, 0.145]				
TL → AC → EIB	0.048 *	0.021	2.308	0.021	[0.010, 0.092]	[0.013, 0.097]				
TL → EIB (total indirect)	0.128 ***	0.031	4.072	0.000	[0.071, 0.191]	[0.073, 0.195]				
TL → EIB (total)	0.189 **	0.073	2.577	0.020	[0.045, 0.330]	[0.045, 0.329]				

Note. * *p* < 0.05; ** *p* < 0.01; *** *p* < 0.001.

**Table 10:** Indirect effect estimates at varying levels of proactive personality.

Levels of Proactive Personality	Psychological Empowerment				Affective Commitment			
	Effect	SE (boot)	BootLLCI	BootULCI	Effect	SE (boot)	BootLLCI	BootULCI
Low level (−SD)	0.0824	0.030	0.0372	0.1352	0.0216	0.016	0.0006	0.0513
Moderate level (0)	0.1027	0.0304	0.0565	0.1571	0.0311	0.0171	0.0074	0.0625
High level (SD)	0.1231	0.0362	0.0676	0.1864	0.0405	0.0211	0.0106	0.0791

Note: BootLLCI_bootstrapped: lower limit confidence interval; BootULCI_bootstrapped: upper limit confidence interval.

**Table 11:** Multi-group analysis for proactive personality.

Relationships	Low PP (n = 153)		High PP (n = 206)		Path Coefficients-Diff	*p*-Value
	Path Coefficients	T Value	Path Coefficients	T Value		
Total effect						
DL → EIB	0.214	1.941	0.430 ***	4.548	−0.216	0.135
Direct effects						
DL → EIB	0.096	0.896	0.307 **	3.115	−0.211	0.144
DL → PE	0.356 ***	3.361	0.352 ***	4.456	0.004	0.964
DL → AC	0.250 **	2.599	0.179 *	2.031	0.071	0.578
PE → EIB	0.267 *	2.339	0.230 **	2.600	0.037	0.789
AC → EIB	0.091	0.852	0.232 **	2.862	−0.141	0.293
Indirect effects						
DL → PE → EIB	0.095	1.856	0.081 *	2.053	0.014	0.843
DL → AC → EIB	0.023	0.748	0.042	1.517	−0.019	0.626

Note: DL = digital leadership, PE = psychological empowerment, AC = affective commitment, PP = proactive personality, EIB = employee innovative behavior. * *p* < 0. 05; ** *p* < 0.01; *** *p* < 0.001.

**Table 12:** 2SLS model testing for endogeneity.

	OLS				IV-2SLS			
	Effect	SE	t	95% CI	Effect	SE	t	95% CI
Digital leadership	0.193 ***	0.055	3.511	[0.085, 0.301]	0.607 *	0.294	2.061	[0.030, 1.184]
Psychological empowerment	0.323 ***	0.063	5.143	[0.200, 0.446]	0.193	0.111	3.812	[−0.024 0.410]
Affective commitment	0.138 **	0.049	2.842	[0.042, 0.234]	0.114 *	0.050	2.613	[0.012, 0.215]
Transformational leadership	0.055	0.055	1.006	[−0.053, 0.163]	−0.157	0.159	−0.993	[−0.468, 0.153]
Hausman’s specification test	F (1, 353) = 2.041, *p* = 0.154							
Over-identifying restriction test	J-statistic = 0.000, *p* = 1.000							
Weak instrument test	Cragg–Donald F statistic = 22.740, Stock–Yoko critical values = 16.38 (size = 10)							

Note. * *p* < 0.05; ** *p* < 0.01; *** *p* < 0.001.

## Data Availability

The raw data supporting the conclusions of this article will be made available by the corresponding authors upon reasonable request.
